# Designing Clean-Label Low-Glycemic Foods: From Starch Structure to Human Glycemic Response

**DOI:** 10.3390/foods15213928

**Published:** 2026-11-02

**Authors:** Methus Chuwech, Sakaewan Ounjaijean, Prapaipit Suwitchayanon, Artorn Anuduang, Yuthana Phimolsiripol, Kongsak Boonyapranai

**Affiliations:** 1 Nutraceutical Research and Innovation Center (Nutri Lab), Research Institute for Health Sciences, Chiang Mai University, Chiang Mai 50200, Thailand; methus.c@cmu.ac.th (M.C.); sakaewan.o@cmu.ac.th (S.O.); artorn.a@cmu.ac.th (A.A.); 2 School of Health Science Research, Research Institute for Health Sciences, Chiang Mai University, Chiang Mai 50200, Thailand; 3 Program in Agriculture, Faculty of Agricultural Production, Maejo University, Chiang Mai 50290, Thailand; prapaipit_swn@mju.ac.th; 4 Faculty of Agro-Industry, Chiang Mai University, Chiang Mai 50100, Thailand; yuthana.p@cmu.ac.th

**Keywords:** physical starch modification, resistant starch, slowly digestible starch, food matrix, starch–polyphenol interactions, D-allulose, INFOGEST, estimated glycemic index, ISO 26642, 3D food printing

## Abstract

Clean-label strategies to lower the glycemic impact of starch-based foods are attracting growing interest, but supporting evidence remains fragmented across starch science, food engineering, digestion methodology, and clinical nutrition. This narrative review, informed by a structured literature search, integrates this evidence into a bottom–up framework linking raw material selection, physical processing, food matrix interactions, in vitro digestion, and human glycemic testing. Using pigmented rice as a model system, it examines how heat–moisture treatment, annealing, ultrasound, extrusion, microwave-assisted processing, and controlled retrogradation reorganize starch, and how hydrocolloids, proteins, lipids, and phenolic compounds reduce enzyme accessibility or enzyme activity. This review makes three contributions. First, mechanistic claims are graded as directly demonstrated, inferred from digestibility data, or hypothetical, and D-allulose–protein conjugation is explicitly treated as a hypothesis. Second, Englyst fractions, INFOGEST kinetics, hydrolysis index, estimated GI, and ISO 26642 human GI are compared as non-interchangeable outputs. Third, an implementation table specifies, for each tier, the design variable, analytical method, evidence required, and principal limitation. The framework is a decision support tool rather than a validated predictive model; formal low-GI claims require human testing, and applications such as 3D-printed foods for older adults require separate validation.

## 1. Introduction

### 1.1. Metabolic Non-Communicable Diseases and the Need for Low-Glycemic Index Diets

According to the 11th edition of the International Diabetes Federation (IDF) Diabetes Atlas, diabetes affected an estimated 589 million adults aged 20–79 years in 2024, corresponding to 11.1% of the global adult population, and this number is projected to reach 853 million by 2050, with type 2 diabetes mellitus (T2DM) accounting for the majority of cases [1]. Dietary patterns rich in rapidly digestible, refined carbohydrates can contribute to large postprandial glucose and insulin excursions, particularly in individuals with impaired insulin sensitivity. Recurrent postprandial hyperglycemia is associated with oxidative stress, inflammatory signaling, β-cell stress, and vascular dysfunction [2,3]. The glycemic index (GI), which ranks carbohydrate-containing foods by their postprandial glucose response relative to a reference food [4], is widely used to characterize carbohydrate quality. Meta-analyses of prospective studies and randomized controlled trials indicate that lower-GI or lower-glycemic-load dietary patterns can improve glycemic control and selected cardiometabolic risk factors, particularly in people with diabetes [5,6].

The rate and site of starch digestion are major determinants of postprandial glycemic response. Englyst and colleagues classified starch operationally, according to its in vitro hydrolysis profile, into rapidly digestible starch (RDS; hydrolyzed within 20 min), slowly digestible starch (SDS; hydrolyzed between 20 and 120 min), and resistant starch (RS; not hydrolyzed after 120 min) under the conditions of the Englyst assay [7]. RS is defined as the fraction of starch and starch degradation products that resists digestion in the small intestine and becomes available for colonic fermentation. It is commonly subdivided into physically inaccessible starch (RS1), native granular starch with enzyme-resistant crystalline organization (RS2), retrograded starch (RS3), chemically modified starch (RS4), and amylose–lipid V-type inclusion complexes (RS5) [8,9]. Starch–polyphenol interactions may also reduce digestibility through inclusion or non-inclusion complexation, although their classification as RS5 remains inconsistent. Shifting a food’s starch profile from rapidly hydrolyzed to slowly digestible or resistant fractions is therefore a central strategy in low-glycemic product design [10].

### 1.2. Chemical Starch Modification and the Clean-Label Context

Chemical modification primarily tailors starch processing and physicochemical functionality, including viscosity, stability, emulsification, and resistance to heat, acid, or shear. Depending on the type and degree of substitution or cross-linking, some modifications may also reduce enzyme accessibility and starch digestibility. Therefore, reduced digestibility should be regarded as a modification-specific outcome rather than a general defining feature of chemically modified starches.

Chemical starch modification introduces substituent groups or cross-links at starch hydroxyl groups. Oxidation, esterification (including acetylation and octenyl succinylation), etherification (such as hydroxypropylation), and phosphate cross-linking are widely used to tailor viscosity, shear and acid tolerance, freeze–thaw stability, retrogradation, and interfacial functionality [11]. Their effects on digestibility vary considerably with the type and degree of modification: some have little effect on amylolysis, whereas sufficiently cross-linked or substituted starches can become less accessible to digestive enzymes and may contribute to RS4. These modified starches are regulated as food additives; in the European Union, relevant products are assigned E-numbers in the E1404–E1452 range and have been re-evaluated by the European Food Safety Authority, which reported no safety concerns for the assessed uses and levels of use [12] (Table 1).

For product developers, the motivation to replace chemically modified starch often stems less from evidence of toxicological risk than from consumer preference, ingredient-list simplicity, and jurisdiction-specific labeling requirements. Consumers may associate additive codes and unfamiliar chemical names with lower perceived naturalness, making clean-label positioning an important market consideration [13]. Physical treatments based on heat, water, pressure, or mechanical energy can alter starch functionality without covalent derivatization [14]. Combined with deliberate control of starch interactions with proteins, lipids, hydrocolloids, and polyphenols, such treatments offer a potential route to lower digestibility while maintaining a comparatively simple ingredient declaration, subject to applicable labeling regulations.

### 1.3. Objectives of This Review

**Figure 1:**
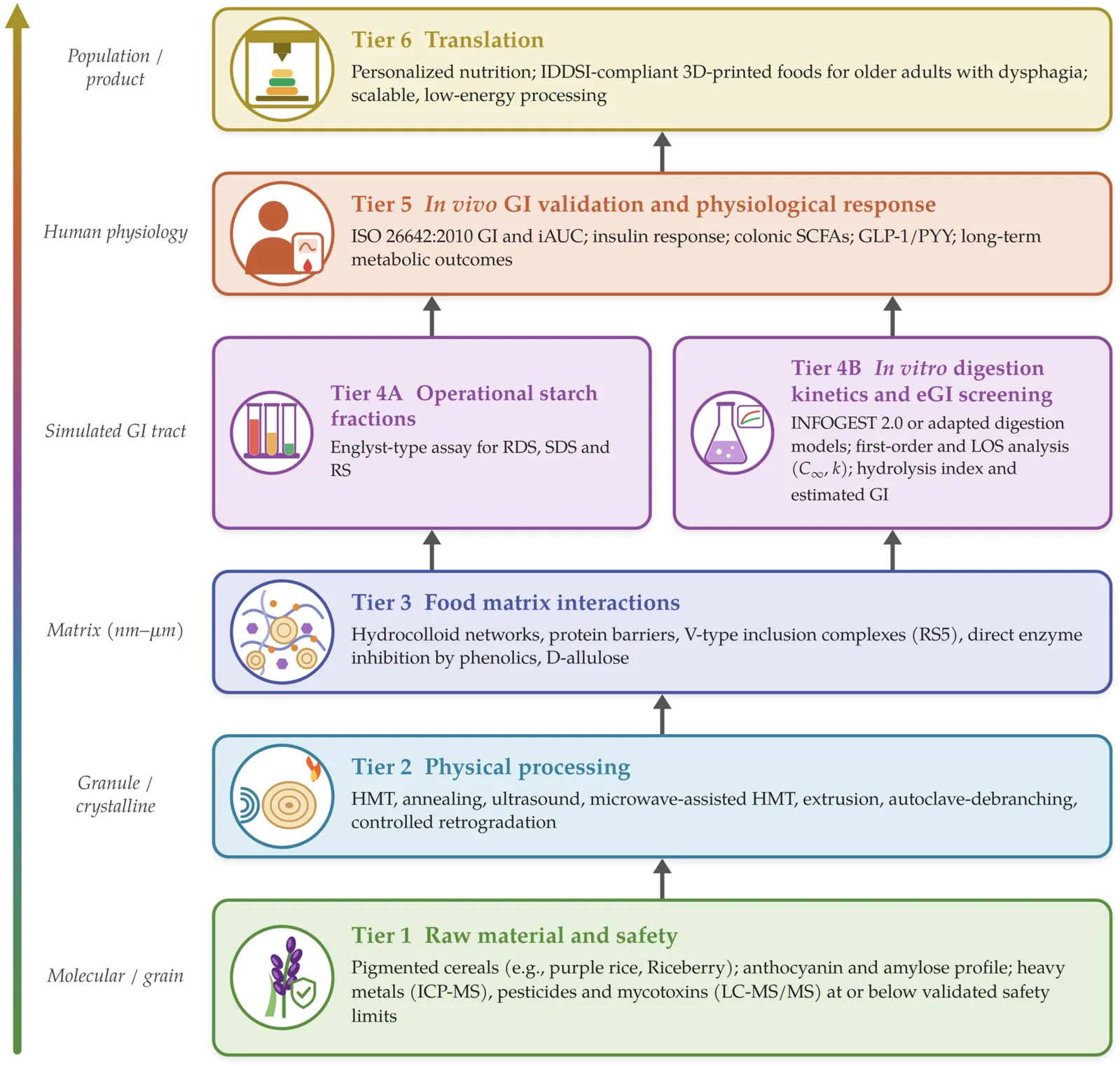
A bottom–up framework for the clean-label design of low-glycemic foods. Each tier provides the structural or analytical foundation for the tier above, moving from raw material selection and safety to translational application. The vertical arrow indicates increasing structural and biological scale, from the molecular/grain level to the population/product level. Tier 4 is separated into two complementary analytical branches: Tier 4A addresses operational starch fractions using Englyst-type assays for rapidly digestible starch (RDS), slowly digestible starch (SDS), and resistant starch (RS), whereas Tier 4B addresses in vitro digestion kinetics and estimated glycemic index (eGI) screening using INFOGEST 2.0 or adapted digestion models, first-order and logarithm-of-slope (LOS) analyses, and hydrolysis index. Both analytical branches provide complementary information for subsequent in vivo validation. Tier 5 focuses on human glycemic index determination and physiological responses, including insulin response and potential colonic and endocrine effects, while Tier 6 emphasizes translation toward personalized nutrition, IDDSI-compliant 3D-printed foods, and scalable processing. The framework is a conceptual decision support tool whose predictive performance has not yet been validated. HMT, heat–moisture treatment; LOS, logarithm of slope; iAUC, incremental area under the curve; SCFAs, short-chain fatty acids; GLP-1, glucagon-like peptide-1; PYY, peptide YY; IDDSI, International Dysphagia Diet Standardisation Initiative.

Evidence on clean-label strategies for lower-glycemic foods is dispersed across food chemistry, materials science, enzymology, and clinical nutrition, while direct links between molecular structure and human glycemic response remain incompletely established. Recent reviews have summarized how physical treatments increase resistant or slowly digestible starch [15,16,17], how non-thermal technologies regulate multiscale starch structure [18], how physical processing of legume starches supports low-GI applications [19], and how starch interacts with non-starch polysaccharides, lipids, proteins, and polyphenols [20,21,22]. These reviews are largely organized by technology or by ingredient class, and most concluded at the level of in vitro digestibility. The present review differs in three respects. First, it follows a single design pathway from raw material to human response, linking each processing or formulation decision to the method that can verify it. Second, it grades the strength of the evidence underlying each proposed mechanism and specifies the type of system from which that evidence derives, from isolated starches to finished foods and human studies (Section 1.4). Third, it treats operational starch fractions, digestion kinetics, estimated glycemic index, and measured human glycemic index as distinct outputs with different uses and limitations. Accordingly, this review develops a bottom–up framework (Figure 1) connecting (i) raw material composition and safety; (ii) physical processing that reorganizes starch at multiple structural scales; (iii) interactions within the food matrix; (iv) operational measurements of starch digestibility and digestion kinetics; and (v) translation to estimated and measured glycemic responses in humans. Particular attention is given to the points at which structural or in vitro evidence supports mechanistic inference but does not yet establish a human GI or clinical effect. Potential applications, including texture-modified foods for older adults, are presented as illustrative translational directions rather than as a universal endpoint of low-glycemic food design. The framework is proposed as a conceptual model and practical decision support tool for researchers and product developers; its predictive performance has not been validated, and its tiers indicate what evidence is required before drawing a conclusion at the next level (Section 7.2).

### 1.4. Literature Search, Study Selection, and Evidence Classification

This article is a narrative review rather than a systematic review or meta-analysis. To make the evidence base transparent and update it during revision, we conducted a structured PubMed search on 27 October 2026 for records published between 1 January 2000 and 26 October 2026, restricting search terms to titles and abstracts. Five search strings were used: (1) starch combined with heat–moisture treatment, annealing, ultrasound, extrusion, microwave treatment, or retrogradation and with digestibility, resistant starch, or slowly digestible starch (1093 records); (2) starch combined with hydrocolloids, non-starch polysaccharides, proteins, lipids, polyphenols, anthocyanins, or allulose and with digestibility, glycemic index, or resistant starch (3278 records); (3) starch combined with in vitro digestion, INFOGEST, hydrolysis index, estimated glycemic index, or the Englyst method (1146 records); (4) glycemic index or postprandial glucose combined with randomized or crossover designs in human participants consuming starch-based foods or rice (185 records); and (5) pigmented, black, or purple rice combined with digestibility, glycemic response, or amylase (81 records). After removal of duplicates, the five searches yielded 4854 unique records. Targeted searches were used for topics not captured by these strings, including global diabetes prevalence, protein–allulose conjugation, process contaminants, and 3D printing for dysphagia diets. We also examined reference lists of key reviews and methodological standards, and retained foundational studies published before 2000 when they define methods or concepts still in use, such as the Englyst classification, the glycemic index, and the hydrolysis index approach.

Retrieved records were screened for relevance to the framework, and studies were selected purposively. Priority was given to (i) studies that reported both structural characterization and starch digestibility under stated conditions; (ii) studies of flours and finished foods in addition to isolated starches; (iii) methodological consensus documents and interlaboratory studies; (iv) human studies that measured glycemic or insulinemic responses using controlled designs; and (v) systematic reviews and meta-analyses where available. Studies were not used when the starch source, treatment conditions, or digestion method were insufficiently described to allow for interpretation. Because the selection was purposive rather than exhaustive, the review does not pool effect sizes, and individual studies are used to illustrate mechanisms and evidence gaps rather than to estimate average treatment effects.

To distinguish established from provisional conclusions, the mechanistic statements in Section 2, Section 3, Section 4, Section 5 and Section 6 are qualified according to three levels of evidence: mechanisms directly demonstrated using structural or biochemical measurements, such as X-ray diffraction (XRD), differential scanning calorimetry (DSC), nuclear magnetic resonance (NMR) spectroscopy, or enzyme kinetic analysis, in the system discussed; mechanisms inferred from changes in starch digestibility or enzyme activity without direct structural confirmation; and hypotheses that are mechanistically plausible but still require direct experimental validation. The evidence source is also specified as an isolated starch or ingredient, a model food system, a finished food, an animal study, or a human study, because a relationship demonstrated in one type of system does not necessarily transfer to another (Section 6.4).

## 2. Raw Material Selection and Safety

### 2.1. Selection of Alternative Raw Materials: Pigmented Rice and Local Cereals

Botanical source establishes the structural and compositional starting point for subsequent processing: amylose content, amylopectin architecture, granule size, crystalline polymorph, endogenous lipids, proteins, and non-starch phytochemicals can all influence starch gelatinization and enzyme accessibility. Pigmented rice (*Oryza sativa* L.) is used here as a model cereal system because it combines a modifiable starch matrix with naturally occurring phenolic compounds. Thai purple-black rice varieties, including *khao kam* and the cross-bred cultivar Riceberry, accumulate anthocyanins in the pericarp and aleurone layers. Cyanidin-3-O-glucoside (C3G) is typically the predominant anthocyanin, with peonidin-3-O-glucoside (P3G) as an important minor component [23,24].

These pigments may reduce carbohydrate digestion through mechanisms beyond antioxidant activity, although the evidence varies in strength by system studied. Akkarachiyasit et al. reported that C3G inhibited pancreatic α-amylase with a half-maximal inhibitory concentration (IC_50_) of 0.30 ± 0.01 mM, while cyanidin-3-galactoside inhibited intestinal sucrase with an IC_50_ of 0.50 ± 0.05 mM; several cyanidin glycosides also showed synergistic inhibition when combined with a low concentration of acarbose [25]. These purified enzyme data demonstrate direct inhibitory capacity, but they do not show that anthocyanins at concentrations found in cooked rice inhibit starch digestion within the grain matrix. Pigmented rice flour is also responsive to physical modification. In low-amylose, polyphenol-rich Riceberry flour, six physical treatments produced distinct in vitro digestion profiles: pregelatinization increased the calculated estimated GI (eGI) to 69.69 ± 2.69, whereas ultrasound treatment produced a lower eGI of 60.77 ± 0.90 and increased polyphenol solubility; annealing and heat–moisture treatment reduced digestion rate and were accompanied by Fourier-transform infrared (FTIR) spectral changes consistent with altered short-range order [26]. Because these results were obtained with flour under a single in vitro protocol, they indicate the direction of processing effects in this raw material rather than the response of a cooked rice product. These findings show that raw material composition and processing should be considered together when tailoring starch digestibility.

### 2.2. Raw Material Safety and Contaminant Control

It is important to establish raw material safety before structural modification, particularly when selecting bran-rich fractions to increase phenolic content. The outer layers of rice that contain anthocyanins may also contain higher concentrations of certain environmental contaminants. Inorganic arsenic in rice varies by geographic origin and can be enriched in bran fractions [27], while soil redox conditions and water management strongly influence arsenic and cadmium accumulation [28]. Elemental analysis by inductively coupled plasma mass spectrometry (ICP-MS), complemented by chromatographic speciation coupled to ICP-MS, where inorganic arsenic must be distinguished, can be used alongside multiresidue pesticide screening based on validated extraction and liquid chromatography–tandem mass spectrometry (LC-MS/MS) methods [29] and appropriate mycotoxin surveillance [30]. Preventive post-harvest drying and humidity-controlled storage remain important for limiting fungal growth. Safety specifications should be defined against applicable maximum limits or maximum residue limits and verified using fit-for-purpose validated methods; where an internal “not quantifiable” target is used, the corresponding method and limit of quantification should be explicitly reported rather than described as “zero residue.”

## 3. Innovative Processing Technologies for Starch Modification

Physical processing can reorganize starch chains through thermal, hydrothermal, dielectric, acoustic, and mechanical energy without deliberate covalent derivatization. The resulting changes depend not on the technology name alone but on botanical source, moisture content, temperature–time history, mechanical energy input, cooling or storage conditions, and the presence of other food constituents. These interacting variables explain much of the apparent inconsistency among published studies, and results obtained with different materials, treatment conditions, and digestion assays cannot be compared directly. Recent syntheses confirm this context dependence: the same treatment can increase SDS under some conditions, and RDS or RS under others [16], and a meta-analysis of ultrasound studies found that RS increased in fruit and cereal starches but decreased in legume and C-type starches [31]. Table 2 therefore summarizes representative technologies, along with the conditions, dominant structural changes, and measured digestion outcomes that define their effects, and Table 3 compares the technologies by efficacy, drawbacks, energy demand, product quality, and industrial applicability.

### 3.1. Heat–Moisture Treatment and Annealing: Effects on Crystallinity and Thermodynamics

Heat–moisture treatment (HMT) exposes starch or flour to restricted moisture, typically below approximately 35% (*w*/*w*), at elevated temperatures for periods ranging from minutes to hours [32,33]; typical conditions and outcomes are summarized in Table 2. Under these conditions, water availability is insufficient for complete gelatinization, and granule morphology is largely retained while molecular mobility within amorphous and interfacial regions permits chain reorganization [34]. Disruption and re-formation of hydrogen-bonding networks can strengthen amylose–amylose and amylose–amylopectin associations, often decreasing swelling power, solubility, and amylose leaching [35]. The magnitude and direction of these changes remain source- and condition-dependent.

DSC commonly shows shifts in gelatinization temperatures and changes in gelatinization range after HMT, while the direction and magnitude of gelatinization enthalpy (Δ*H*) depend on botanical source and treatment severity [32,36]. XRD may reveal changes in relative crystallinity or polymorphic organization; some tuber starches show B- to A-like transitions, whereas lipid-containing systems may develop V-type reflections [32]. In separated wheat starch granules, HMT produced A+V-type patterns, and B-type granules responded more strongly than A-type granules [37]. Importantly, relative crystallinity does not consistently increase after HMT. In rice starch blended with xanthan or locust bean gum, HMT decreased measured crystallinity, while RS increased from 8.42 ± 0.39% in native starch to 38.36 ± 3.69% in treated starch–hydrocolloid systems, accompanied by reductions in hydrolysis parameters and calculated eGI [38]. These observations show that reduced digestibility can arise from multiple structural features, including restricted swelling, altered molecular mobility, local short-range order, granule or matrix barriers, and long-range crystalline organization. Crystallinity should therefore not be used as a single proxy for enzyme resistance. Treatment severity must also be optimized in the material that will actually be consumed: in whole highland barley flour, HMT reorganized starch–protein structures and altered both noodle quality and starch digestibility, with structural order enhanced at 80 °C but disrupted at 120 °C [39].

Annealing (AN) incubates starch in excess water, generally above 40% and often above 60% (*w*/*w*), at temperatures below the onset of gelatinization for hours to days. Rather than causing extensive granular disruption, AN promotes limited molecular mobility and improved alignment or perfection of pre-existing double-helical regions, typically narrowing the gelatinization temperature range while preserving granule morphology [40]. Its effects on digestibility are often smaller than those produced by HMT, although the response varies among starch sources. AN and HMT can also be applied sequentially to generate complementary structural effects, but the resulting digestibility should be established empirically rather than assumed to be additive [35].

**Table 2:** Physical and enzyme-assisted clean-label technologies for modulating starch digestibility: typical processing conditions, dominant structural effects, and representative outcomes.

Technology	Typical Conditions	Dominant Structural Effects	Representative Outcome
Heat–moisture treatment (HMT)	<35% moisture; 84–120 °C; 15 min to 16 h	Chain rearrangement; increased gelatinization temperatures; reduced swelling and amylose leaching; polymorphic changes; relative crystallinity may increase, decrease, or remain unchanged depending on starch source and treatment conditions	Rice starch with xanthan or locust bean gum: RS increased from 8.42% to up to 38.36%, with lower eGI [38]. HMT rice improved glucose tolerance in high-fat-fed mice [41]
Annealing (AN)	Excess water; below onset gelatinization temperature; hours to days	Improved alignment of existing double helices; narrower gelatinization temperature range; granule morphology largely retained	Lower digestion rate of Riceberry flour, accompanied by an increased FTIR-derived indicator of local short-range order [26]
Extrusion with controlled cooling	High temperature, short residence time; high shear; outcome strongly dependent on moisture, specific mechanical energy, temperature profile, formulation, and post-extrusion cooling	Depending on processing severity, starch may undergo gelatinization, molecular degradation, rearrangement, retrogradation, or amylose–lipid complex formation	Digestibility is strongly process-dependent. Severe gelatinization and depolymerization can increase RDS, whereas inclusion of lipids/emulsifiers and controlled post-extrusion cooling or tempering can promote V-type complex formation and/or retrogradation, thereby reducing starch digestibility [42,43]. Enzymatic extrusion of oat flour increased RS from 6.87% to 31.99% [44]
Ultrasound (US), alone or with HMT	Aqueous suspension; minutes	Cavitation-induced surface erosion, pores and fissures; possible chain scission; when combined with HMT, may promote molecular rearrangement	Riceberry flour eGI 60.77 (US) vs. 69.69 (pregelatinized) [26]; in purple rice starch, the FTIR 1047/1022 cm^−1^ ratio increased from 1.29–1.45 after HMT to 1.38–1.64 after HMT–US, indicating greater local short-range order, while overall relative crystallinity decreased [45]. Meta-analysis: direction of RS change depends on starch source and crystal type [31]
Microwave-assisted HMT	Dielectric heating; minutes	Rapid volumetric heating; accelerated molecular rearrangement; potentially heterogeneous heating if moisture or field distribution is uneven	Substantially shorter processing time than conventional HMT; reproducibility and heating uniformity remain important scale-up considerations [14]. Wheat starch RS 36.89% after 1.5 h, decreasing at 2 h [46]
Debranching–autoclaving with polyphenol	Pullulanase treatment; autoclaving at 105 °C; cooling at 4 °C	Formation of shorter linear chains followed by recrystallization and/or V-type complex formation; B+V-type diffraction pattern	Chinese yam starch–tea polyphenol complex: RS 56.25%, RDS 22.92%, with a V-type reflection at 19.5° 2θ [47]

Abbreviations: HMT, heat–moisture treatment; AN, annealing; US, ultrasound; RS, resistant starch; RDS, rapidly digestible starch; eGI, estimated glycemic index.

### 3.2. Emerging Physical Processing: Extrusion, Ultrasound and Microwave Treatment

Extrusion combines heat, pressure, shear, and short residence time, and can simultaneously induce starch gelatinization, molecular fragmentation, mixing, and complexation (Table 2). Under high-energy, low-moisture conditions, increased gelatinization and depolymerization often increase enzyme accessibility and rapidly digestible starch [42]. However, extrusion is better viewed as a structure-generating platform than as an intrinsically high- or low-digestibility process. The final digestion profile depends on specific mechanical energy, feed moisture, thermal history, formulation, and post-extrusion cooling. Inclusion of suitable lipids or emulsifiers can promote amylose–lipid V-type complexation, while controlled cooling or tempering can increase retrogradation of solubilized linear chains [43]. Combining extrusion with enzymatic debranching exploits this principle: pullulanase-assisted extrusion of whole oat flour increased RS from 6.87% to 31.99% through complexation with endogenous lipids [44], and a two-step extrusion–debranching–complexing process with lauric acid yielded RS of up to 38.34% in corn starch [48]. Such continuous processes are attractive for scale-up, but enzyme use must be considered when aiming for clean-label positioning.

High-intensity ultrasound generates acoustic cavitation in aqueous starch systems (Table 2). Collapse of cavitation bubbles produces localized shear, micro-jets, surface erosion, pores, fissures, and, under sufficiently intense conditions, molecular scission [49]. These changes can increase digestibility by exposing internal regions or, when combined with hydrothermal treatment, facilitate subsequent molecular rearrangement. In purple rice starch, ultrasound after HMT dispersed HMT-induced aggregates and increased the FTIR 1047/1022 cm^−1^ band ratio from 1.29–1.45 to 1.38–1.64, consistent with increased local short-range order, while relative crystallinity decreased and the A-type diffraction pattern was retained [45]. Microwave-assisted HMT provides rapid volumetric heating through dielectric interactions and can markedly shorten processing time [14]. In rice starch, microwave-assisted HMT combined with avocado oil reduced digestibility and eGI under milder time–temperature conditions than autoclave HMT [50]; in quinoa starch, moisture content determined whether short-range order and crystallinity increased [51]; and in wheat starch, RS increased to 36.89% after 1.5 h of microwave-assisted HMT but declined when treatment was extended to 2 h [46], indicating an optimum beyond which excess heating disrupts ordered structure. Depending on moisture distribution, peak temperature, and cooling history, such treatment may promote rearrangement or retrogradation, but non-uniform heating and scale-dependent temperature gradients remain important engineering constraints.

Two further approaches complement these physical treatments. Enzymatic debranching with pullulanase increases the population of shorter linear chains that can subsequently recrystallize or form inclusion complexes. When Chinese yam starch was debranched and autoclaved at 105 °C in the presence of tea polyphenols, the resulting material showed B+V-type diffraction features, a new reflection near 19.5° 2θ, an increase in relative crystallinity from 14.54% to 20.07%, and an RS content of 56.25 ± 1.37% with RDS of 22.92 ± 0.14% [47]. Controlled retrogradation is another route to reduced digestibility: amylose can reassociate relatively rapidly after gelatinization, whereas amylopectin recrystallization proceeds more slowly and contributes strongly to staling [52], with recrystallization kinetics often described using Avrami-type models [52,53]. Retrograded structures are intrinsically less susceptible to amylolysis, and some retrograded starch preparations can additionally interact with α-amylase and reduce hydrolysis of coexisting gelatinized starch [54].

### 3.3. Structural Characterization

Because no single method captures the multiscale structural changes induced by processing, orthogonal characterization is required. XRD primarily reports long-range crystalline order and polymorphic features; V-type amylose inclusion complexes commonly show reflections near 7.5°, 13°, and 20° 2θ [43]. Attenuated total reflectance (ATR)–FTIR is sensitive to local molecular organization near the granule surface, and ratios such as 1047/1022 cm^−1^ are widely used as empirical indicators of short-range order, but they should not be interpreted as direct measures of crystallinity [55,56]. Solid-state 13C cross-polarization magic-angle spinning (CP/MAS) NMR can provide complementary information on glycosidic conformations and single- versus double-helical environments [57], while DSC measures the thermal stability and enthalpy associated with ordered domains [36]. Convergent evidence from these techniques, ideally combined with microscopy or scattering where appropriate, provides a stronger mechanistic basis than any individual measurement [56].

### 3.4. Industrial Scale-Up and Engineering Constraints

Translating starch modification technologies from the lab to manufacturing requires control of both process engineering and product variability. Batch HMT systems can suffer from slow or uneven heat and moisture transfer, producing heterogeneous treatment severity, while prolonged holding increases energy demand. Continuous hydrothermal systems, microwave-assisted heating, and extrusion may shorten processing time or improve throughput, but researchers must verify their effects on RS, slowly hydrolyzed starch, sensory quality, and batch-to-batch reproducibility at scale rather than extrapolating from small laboratory preparations [14,42]. Reporting process variables and specific energy use per kilogram of product alongside structural and digestibility outcomes would support more meaningful techno-economic and environmental comparisons.

Practically, the technologies differ more in their operating windows and engineering constraints than in any single digestibility outcome (Table 3). HMT and AN require only water and heat and are compatible with existing conditioning and drying equipment, but long holding times, particularly for AN, increase energy use, and the subsequent drying step adds cost. Microwave-assisted HMT shortens treatment times and can lower energy input [50], but it is sensitive to moisture and field distribution and can over-process starch if exposure is prolonged [46]. Ultrasound works best in dilute aqueous suspensions, which limits throughput and increases the drying load, and its outcomes depend strongly on the starch source [31]. Extrusion is already continuous and widely used industrially [58], but high specific mechanical energy and low feed moisture can increase RDS and Maillard-derived process contaminants such as acrylamide and 5-hydroxymethylfurfural [59]. Product quality must be evaluated alongside digestibility, because reduced swelling, altered pasting, firmer texture, color change, or loss of aroma-active and phenolic compounds can limit consumer acceptability [39,58]. Comparative techno-economic and life-cycle data remain scarce; reporting specific energy consumption per kilogram of product, water use, and yield, along with structural and digestibility outcomes, would allow us to rank technologies beyond laboratory efficacy.

Preclinical evidence suggests that HMT-induced structural changes may translate into metabolic effects in vivo. In C57BL/6 mice fed a high-fat diet for three months, replacement of untreated rice with HMT rice improved oral glucose tolerance and reduced serum cholesterol and body-weight gain; these changes were accompanied by altered serum lysophospholipids and fecal oxidized phospholipids and bile acid derivatives [41]. Although these findings support biological plausibility, they should not be interpreted as confirmation of comparable effects in humans, for whom controlled trials of physically modified staple foods remain limited. In this evidence classification, this study provides animal evidence that the processed food alters glucose tolerance, but it did not isolate the responsible structural features.

**Table 3:** Comparative assessment of physical starch modification technologies for clean-label, lower-glycemic applications: effect on digestibility, principal drawbacks, energy and cost considerations, product quality effects, and industrial applicability.

Technology	Typical Effect on Digestibility (Evidence Source)	Principal Drawbacks	Energy and Cost Considerations	Product Quality Effects	Industrial Applicability
HMT	Usually lowers digestion rate and may increase RS or SDS; magnitude and direction depend on source, moisture, and temperature (isolated starches, flours; few finished foods) [32,38,39]	Slow or uneven heat and moisture transfer in batch systems; over-treatment can disrupt ordered structure	Moderate to high: heating for minutes to hours, moisture adjustment, and drying	Reduced swelling and viscosity; firmer texture; possible color change	High for flour and ingredients; compatible with existing conditioning and drying equipment
AN	Generally smaller reductions in digestibility than HMT (isolated starches, flours) [26,35,40]	Very long incubation; excess water must be removed; microbial control needed during incubation	High water use and drying load; long residence times	Narrower gelatinization range; modest functional change	Moderate; best suited to starch or flour ingredients
Extrusion (with lipids, debranching, or controlled cooling)	Severe conditions increase RDS; lipids, debranching, or cooling can increase RS through V-type complexes and retrogradation (flours, model systems) [42,43,44,48]	Depolymerization under severe conditions; Maillard-derived contaminants at low moisture and high temperature [59]	Continuous and efficient per unit product, but high mechanical energy input	Expansion, crispness, color, and flavor change; texture strongly formulation-dependent	Very high; already used for snacks, cereals, and pasta [58]
Ultrasound	RS may increase or decrease depending on starch source and crystal type (isolated starches; meta-analysis) [31,45,49]	Requires dilute suspensions; possible chain scission; limited penetration in viscous systems	High electrical energy per unit product at low solids; drying required	Usually, minor sensory effect at ingredient level; may increase solubility	Low to moderate; mainly pilot scale or combined with other treatments
Microwave-assisted HMT	Can reduce digestibility rapidly, with an optimum treatment time (isolated starches) [46,50,51]	Non-uniform heating and hot spots; scale-dependent field distribution	Shorter treatment time and potentially lower energy than conventional HMT	Similar to HMT; risk of localized over-processing	Moderate; uniformity of continuous systems must be validated
Controlled retrogradation (cooking, cooling, storage)	Increases RS3 and can lower postprandial glucose of cooled staples (finished foods; human studies) [52,54,60]	Partly reversible on reheating; staling and hardening	Low energy for cooling, but storage time and cold-chain costs	Hardening, staling, reduced palatability	High for chilled and ready-to-eat products; requires storage control
Enzymatic debranching (with lipids or polyphenols)	Large RS increases through recrystallization or complexation (isolated starches, flours) [44,47]	Enzyme use and labeling considerations; additional process steps	Enzyme cost; incubation and autoclaving steps	Altered solubility and viscosity	Moderate; feasible where enzyme processing is established

Qualitative comparison based on the cited studies and reviews [14,15,33,35,42,58]. Energy and cost categories are indicative because comparable techno-economic data are rarely reported. HMT, heat–moisture treatment; AN, annealing; RS, resistant starch; RDS, rapidly digestible starch; SDS, slowly digestible starch; RS3, retrograded resistant starch.

## 4. Synergistic Effects in the Food Matrix: Macromolecular Interactions

In real foods, starch is digested within a dynamic matrix of water, proteins, lipids, fibers, and phytochemicals whose organization is shaped by processing and storage. Digestion rate therefore depends not only on the amylose-to-amylopectin ratio but also on granule integrity, porosity, cell wall encapsulation, phase separation, viscosity, interfacial barriers, and molecular interactions among matrix components [61]. Low-glycemic design should thus be considered a multiscale food structure problem rather than a starch composition problem alone. Table 4 summarizes the major matrix interactions discussed below.

Two classes of matrix effects should be distinguished because they are demonstrated and tested in different ways. The first is reduced enzyme accessibility: matrix components restrict starch swelling and gelatinization, slow α-amylase diffusion to the substrate, or physically encapsulate starch without altering the enzyme itself. Evidence for this mechanism comes from microscopy, swelling and rheological measurements, and comparisons between the intact matrix and isolated starch; the effect typically diminishes when the matrix is disrupted. The second is a direct effect on digestive enzymes: free or released components, most notably phenolic compounds, bind to α-amylase or α-glucosidase and reduce catalytic activity. This effect is demonstrated with purified enzymes and kinetic analysis and can act on fully accessible starch. Retrograded starch that binds α-amylase [54] and polyphenols that both complex with starch and inhibit enzymes [69] can act through both routes, so experiments should include controls that separate them, for example by removing unbound polyphenols before digestion or by adding the putative inhibitor to a starch that has not been co-processed with it.

### 4.1. Starch–Polysaccharide Interactions and Rheological Behavior

Non-starch polysaccharides such as xanthan gum, guar gum, locust bean gum, konjac glucomannan, and pectin interact with starch through water competition, hydrogen bonding, phase behavior, and changes in continuous-phase rheology [63,71]. Depending on the starch–hydrocolloid combination, these interactions can alter granule swelling, amylose leaching, pasting, and gelation. Increased viscosity can also slow convective mixing and reduce the frequency of enzyme–substrate encounters during digestion. In vivo, viscous fibers can attenuate postprandial glycemia through mechanisms that may include altered gastric emptying, luminal mixing, and nutrient transport. Early human studies demonstrated that the glycemia-lowering effect of several fiber preparations was strongly related to viscosity [62].

Some hydrocolloids can also form surface-associated layers or continuous hydrated networks that restrict starch swelling or enzyme access. In rice starch systems containing xanthan or locust bean gum, microscopy showed coating-like associations around starch granules. After HMT, these systems exhibited lower peak viscosity and breakdown, delayed gelatinization, and lower storage (G’) and loss (G″) moduli than native starch, while RS increased markedly and FTIR indicated altered local order without evidence of new covalent bonds [38]. Notably, these observations indicate that digestion resistance does not necessarily require formation of a mechanically stronger gel. In this system, restricted swelling, water redistribution, and reduced enzyme accessibility may be more informative than bulk rheological strength alone. Rheology should therefore be interpreted together with hydrated microstructure and digestibility measurements rather than used as a direct proxy for starch resistance. This study provides direct structural evidence in a model system; whether the same coating and swelling restriction persist in cooked rice products remains to be shown.

### 4.2. Starch–Protein Interactions and Protein–Sugar Conjugation as Research Hypothesis

Proteins can influence starch digestion through hydrogen bonding, hydrophobic interactions, electrostatic effects, competition for water, and formation of matrix domains that restrict starch swelling or enzyme access. In rice flour, removal of endogenous proteins and lipids increased starch digestibility, supporting a protective role for native matrix components [64], while potato starch–protein blends also showed lower in vitro digestibility than starch alone [65]. The magnitude of this effect depends on protein type, denaturation state, food microstructure, and processing history. During gastrointestinal digestion, gastric proteolysis can partially disrupt protein-based barriers, potentially increasing subsequent pancreatic α-amylase access; therefore, protein-mediated protection should be evaluated using sequential digestion rather than inferred from undigested food structure alone.

Protein–sugar conjugation, in contrast, should be presented as a research hypothesis rather than as an established clean-label strategy. Experimental evidence is limited to the conjugation step and its effects on protein properties. Maillard-type conjugation alters protein conformation, solubility, interfacial behavior, and thermal properties [66]. D-Allulose (D-psicose), the C-3 epimer of D-fructose, is a reducing ketohexose that can participate in Maillard chemistry and has a low energy value relative to sucrose [72,73], and it has been conjugated with soy protein hydrolysate and pumpkin seed protein, modifying antioxidant, enzyme-inhibitory, solubility, and emulsifying properties [67,68]. What has not been demonstrated is that such conjugates, when present in a starch-based food, slow starch hydrolysis, persist through gastric proteolysis, or lower postprandial glycemia. The proposed mechanism, that conjugated proteins form more persistent barriers around starch granules, therefore remains a hypothesis that requires direct experimental validation. Testing it would require (i) confirmation and quantification of conjugation, for example by loss of free amino groups, glycation markers, and molecular weight changes; (ii) comparison of starch digestion in matrices containing native protein, conjugated protein, and an unreacted mixture of protein and allulose under sequential gastric and intestinal digestion; (iii) microscopy of protein–starch organization before and after pepsin exposure; (iv) quantification of potentially undesirable Maillard reaction products; and (v) human glycemic testing of any lead formulation. Because free allulose itself attenuates postprandial glucose when co-ingested (Section 6.3), human studies would also need to separate the effects of the conjugate from those of residual free allulose.

### 4.3. Food Structure Dynamics at the Microscale

Hydrolysis of intact or partially gelatinized starch by α-amylase is an interfacial process involving enzyme transport to the substrate, adsorption, formation of a productive enzyme–substrate complex, and catalysis [61,74]. Granule morphology therefore strongly influences apparent digestion kinetics. Many cereal starch granules, including certain maize and wheat granule populations, contain surface pores or internal channels that can facilitate enzyme penetration, whereas potato starch generally has a smoother surface and is more commonly eroded from the exterior [61]. Because pore density, granule damage, and internal architecture vary with botanical source and processing, interventions that alter accessible surface area, hydration, diffusion, or productive enzyme binding can markedly change digestion rate.

**Figure 2:**
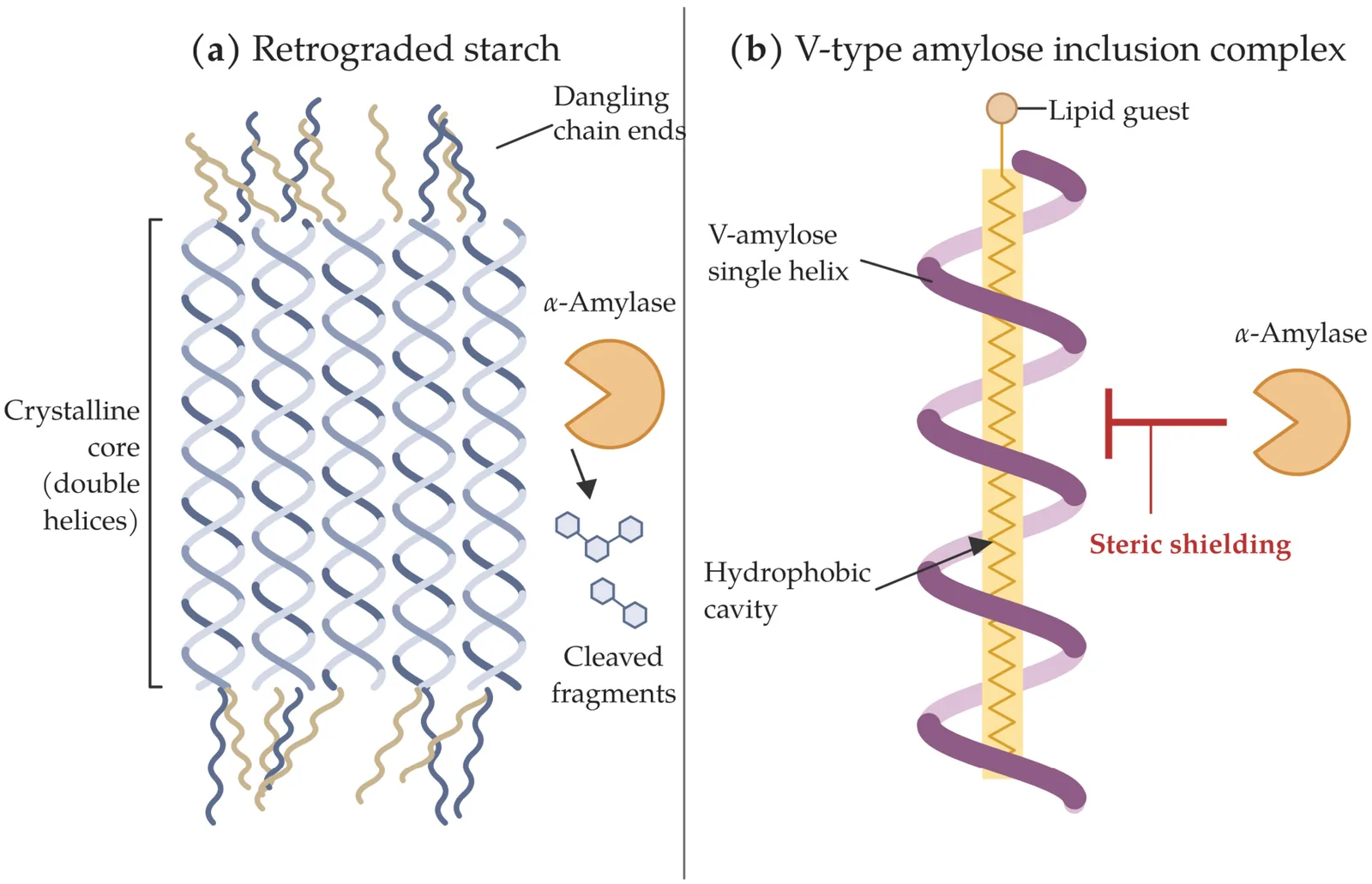
Structural basis of differential α-amylase susceptibility. (**a**) Retrograded starch: double helices aggregate into crystalline core, whereas dangling chain ends and imperfectly packed segments at periphery remain accessible to α-amylase and are hydrolyzed to soluble fragments. (**b**) V-type amylose inclusion complex: lipid guest occupies hydrophobic cavity of amylose single helix, and compact helical conformation sterically shields glycosidic bonds from enzyme. Schematics based on concepts described in [9,43,70].

At the molecular scale, amylose heated in the presence of suitable hydrophobic ligands can adopt a left-handed single-helical conformation with a non-polar cavity. Fatty acids and monoglycerides can occupy this cavity to form V-type amylose–lipid inclusion complexes, conventionally classified as resistant starch type 5 (RS5) [43,70]. Certain phenolic compounds may also form amylose inclusion complexes when their molecular size and hydrophobicity are compatible with the helical cavity, while others interact through non-inclusion hydrogen bonding or surface association [75]. These starch–polyphenol interactions can reduce amylolysis by increasing local structural organization or limiting enzyme access, and released phenolics may additionally inhibit digestive enzymes [76]. Nevertheless, three types of evidence should be distinguished: evidence of complex formation, such as V-type diffraction, altered thermal transitions, or spectroscopic shifts measured after removing unbound polyphenol; evidence of enzyme inhibition, obtained with purified enzymes and kinetic analysis; and evidence of reduced digestion in a complete food matrix. Reduced glucose release from a starch–polyphenol system does not by itself establish complex formation, because the same outcome can arise from surface adsorption, substrate shielding, or inhibition by free polyphenols [22,69]. Because the structural basis differs among ligands, starch–polyphenol complexes should not be categorized uniformly as RS5 without direct evidence of a V-type inclusion structure [69] (Figure 2).

## 5. In Vitro Digestibility, Enzyme Kinetics, and Bioactivities

### 5.1. Inhibition of α-Amylase and α-Glucosidase by the Modified Matrix

A modified food matrix can slow carbohydrate digestion through at least two mechanistically distinct routes: by restricting enzyme access to starch and by directly reducing digestive-enzyme activity. Phenolic compounds released from the matrix can inhibit salivary or pancreatic α-amylase and intestinal α-glucosidases, with potency strongly influenced by hydroxylation pattern, glycosylation, molecular size, and hydrophobicity [77,78]. Enzyme kinetic analysis can distinguish competitive, non-competitive, uncompetitive, or mixed behavior and estimate inhibition constants for binding to free enzyme and/or enzyme–substrate complexes [76,79]. When sufficient data are available, directly fitting candidate kinetic models with nonlinear regression is preferable to relying solely on Lineweaver–Burk linearization, which can magnify experimental error. Inhibition mechanisms should therefore be established for each phenolic–enzyme–substrate system rather than generalized across polyphenols.

Retrograded starch illustrates why substrate structure and direct enzyme effects should be distinguished. Reassociated amylose and amylopectin domains are intrinsically less susceptible to hydrolysis because of tighter molecular packing and reduced accessibility; selected retrograded starch preparations have also been shown to bind α-amylase and reduce hydrolysis of accompanying gelatinized starch [54]. Anthocyanins such as C3G provide a different mechanism by directly inhibiting α-amylase and intestinal sucrase, with reported synergy at low acarbose concentrations [25]. In whole foods, however, phenolics partition among starch, proteins, lipids, aqueous phases, and digestive enzymes. Consequently, inhibition measured with purified enzymes does not directly predict the magnitude of effect within a complex food matrix [76]. Half-maximal inhibitory concentrations obtained with purified enzymes should therefore be interpreted as evidence of inhibitory capacity, not as evidence of a glycemic effect.

### 5.2. Starch Digestion Kinetics and Operational Digestibility Fractions

Major differences in enzyme source, activity, pH, mixing, and phase duration have hindered comparisons of digestion data across laboratories. The INFOGEST network developed a harmonized static gastrointestinal protocol [80], subsequently refined as INFOGEST 2.0 [81], to standardize oral, gastric, and intestinal digestion conditions. The protocol specifies sequential exposure to salivary α-amylase, gastric pepsin and lipase, and intestinal pancreatin and bile under controlled pH and temperature conditions [81] (Table 5). For starch-focused applications, however, pancreatin is standardized primarily by trypsin activity, and α-amylase activity can vary markedly among batches; therefore, intestinal α-amylase activity should be independently measured and reported. An interlaboratory study within the INFOGEST network found substantial between-laboratory variation with the widely used single-point α-amylase assay at 20 °C, whereas an optimized four-time-point assay at 37 °C reduced interlaboratory coefficients of variation to 16–21%; activity measured at 37 °C was approximately 3.3-fold higher than at 20 °C [82]. Because the apparent rate of hydrolysis depends on the enzyme-to-substrate ratio, differences in actual α-amylase activity change the absolute values of fitted kinetic parameters and of the indices derived from them, and an enzyme dose that is too high or too low can compress or exaggerate differences between treatments. Treatment effects are therefore most interpretable when control and treated samples are digested in the same run with the same measured enzyme activity. Importantly, do not equate INFOGEST-derived hydrolysis kinetics directly with Englyst RDS, SDS, and RS fractions unless those fractions are determined using an appropriate operational method. Static INFOGEST also does not reproduce gastric emptying, peristaltic mixing, intestinal absorption, or hormonal feedback; semi-dynamic or dynamic models can address selected aspects of these limitations [83,84].

Under defined assay conditions, starch hydrolysis curves are often approximated using a first-order model [90], although many intact or heterogeneous plant foods display more than one kinetic phase:

*C_t_* = *C*_∞_(1 − e^−*kt*^), (1)

where *C_t_* is the percentage of starch hydrolyzed at time *t*, *C*_∞_ is the model-estimated asymptotic extent of hydrolysis, and *k* is the apparent first-order rate constant under the specified assay conditions. Because *C*_∞_ is estimated from the fitted model rather than measured directly at an infinite endpoint, its value is sensitive to the duration and information content of the digestion curve. The logarithm-of-slope (LOS) transformation can diagnose kinetic phases and estimate first-order parameters [90,91].

(2) $$\ln\left(\frac{dC}{dt}\right)=\ln\left(C_{\infty }k\right)-\mathrm{kt}$$

**Figure 3:**
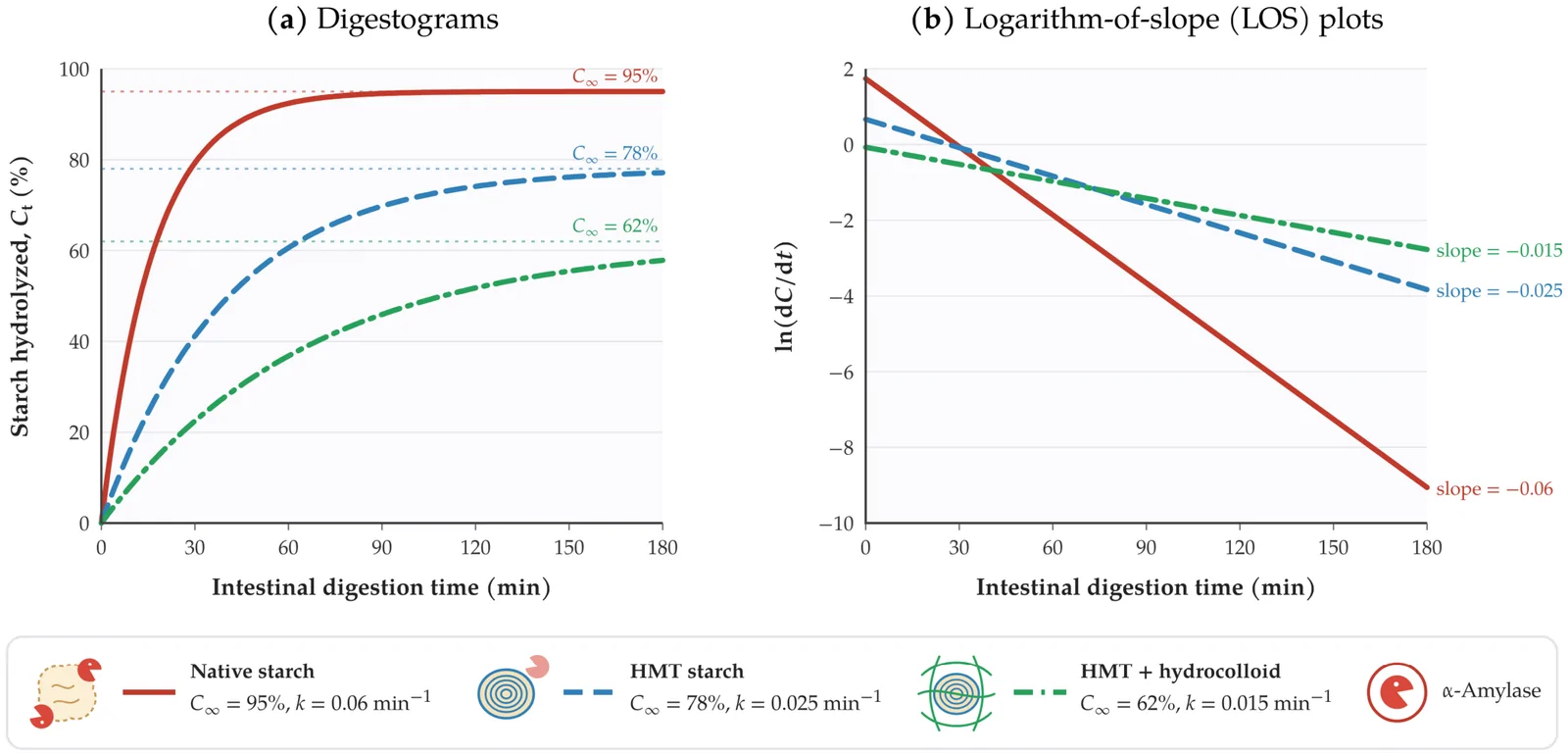
Illustrative first-order digestion kinetics. (**a**) Digestograms simulated with Equation (1) [90] for native starch, heat–moisture-treated (HMT) starch, and HMT starch combined with a hydrocolloid, using representative parameter values chosen to illustrate differences in hydrolysis rate and model-estimated digestion extent. (**b**) Corresponding logarithm-of-slope (LOS) plots according to Equation (2) [91,92], in which the slope is related to the apparent first-order rate constant, *k*. The curves are illustrative and should not be interpreted as operational RDS, SDS, or RS fractions.

In an ideal single-phase first-order process, the LOS representation yields a slope related to −*k* and an intercept related to *kC*_∞_. Deviations from linearity or distinct linear regions can reveal sequential phases with different apparent rate constants, as observed in many cooked plant tissues and structurally heterogeneous foods [91,92] (Figure 3). Processing may reduce *k*, *C*_∞_, or both, but these parameters should be interpreted mechanistically rather than assigned directly to Englyst fractions. A lower *k* is consistent with slower hydrolysis of the enzyme-accessible fraction, whereas a lower fitted *C*_∞_ suggests that a larger fraction remains inaccessible or very slowly accessible within the experimental time window. Neither parameter is a direct quantitative surrogate for SDS or RS unless those fractions are independently determined using an appropriate operational assay.

Some in vitro GI prediction approaches normalize the area under a starch-hydrolysis curve (AUC) to that of a reference food to generate a hydrolysis index (HI) [85]. In the original Goñi approach, white bread was used as the reference. HI is therefore an assay- and reference-dependent empirical index rather than a direct physiological measurement:

(3) $$\mathrm{HI}=\frac{{\mathrm{AUC}}_{\mathrm{sample}}}{{\mathrm{AUC}}_{\mathrm{reference}}}\times 100,$$

The HI can then be entered into an empirical regression equation to obtain an estimated glycemic index (eGI), for example, the equation originally proposed by Goñi et al. [85]:

eGI = 39.71 + 0.549 × HI.(4)

An alternative relationship, eGI = 8.198 + 0.862 × HI, was proposed by Granfeldt et al. [86]. They calibrated such equations using different experimental procedures, reference materials, and food sets, so they are not interchangeable. An eGI value should always be reported together with the digestion method and regression equation used, and any claim of predictive validity should be supported by calibration against measured human GI in the relevant food category (Section 6.1).

The approaches discussed in this section and in Section 6 therefore answer different questions (Table 5). Englyst-type assays classify starch into operational fractions based on glucose released at fixed times under defined pancreatic α-amylase and amyloglucosidase conditions [7]. INFOGEST-based or adapted protocols simulate sequential oral, gastric, and intestinal phases and yield a hydrolysis time course from which kinetic parameters can be fitted [81]. HI and eGI convert such a time course into a single value by reference to a standard food and an empirical regression equation [85,86]. Human GI testing measures the integrated glycemic response in vivo (Section 6.2). Several practical rules follow. RDS, SDS, and RS cannot be inferred directly from fitted INFOGEST parameters, and *k* or *C*_∞_ cannot be converted into Englyst fractions. Enzyme source and activity, substrate concentration, mixing, particle size, sample preparation, sampling duration, and the glucose analysis method all influence in vitro results. HI and eGI values should be compared across studies only when the digestion protocol, reference food, and regression equation are the same or have been shown to give comparable results. Finally, in vitro data are best used for formulation screening and mechanistic investigation, whereas GI classification requires standardized human testing.

### 5.3. Safety and Bioactivities After Thermal Processing

Safety considerations can be divided into general concerns that apply to any processed starch-based food and specific concerns associated with particular treatments or ingredients. Physical processing avoids the deliberate use of chemical derivatizing reagents but does not eliminate the need to evaluate composition and process safety. General concerns include raw material contaminants (Section 2.2), microbiological safety during moisture conditioning, cooling, and storage, and regulatory requirements for the final food, which differ among jurisdictions. Thermal treatment in the presence of reducing sugars and amino groups can generate a broad range of Maillard reaction products. For emerging strategies such as allulose–protein conjugation, therefore, it is important to optimize process intensity not only for functionality but also to minimize potentially undesirable advanced-stage products, including selected advanced glycation end-products where analytically and biologically relevant. Evidence that allulose has been tolerated in human feeding studies [93] supports the ingredient itself but does not replace safety characterization of a thermally processed allulose-containing matrix. A practical safety dossier should combine raw material contaminant control with fit-for-purpose assessment of process-induced compounds in the final product.

Specific concerns differ by technology and ingredient. High-temperature, low-moisture extrusion and roasting can generate acrylamide, 5-hydroxymethylfurfural, and α-dicarbonyl compounds. In extruded rice flour systems, feed moisture was the dominant factor controlling acrylamide and 5-hydroxymethylfurfural formation [59], and in an oat paste meeting International Dysphagia Diet Standardisation Initiative (IDDSI) Level 3 criteria, roasting at 160 °C produced 507.4 μg/kg acrylamide and reduced SDS, whereas roasting at 90–110 °C produced no detectable acrylamide [94]. Ultrasound can generate radicals in aqueous media and may promote lipid or phenolic oxidation [49]; microwave heating can create localized hot spots; and enzymatic debranching requires food-grade enzyme preparations whose status as processing aids must be confirmed under applicable regulations. For ingredients, high intakes of allulose or viscous hydrocolloids may cause gastrointestinal discomfort, and allulose’s regulatory status differs by jurisdiction. These specific hazards should be assessed in the final product rather than inferred from the safety of the starting ingredients.

Retention of bioactive phytochemicals is a second design constraint. Anthocyanins are heat-labile: cooking black rice reduced C3G by 65.4% on a gas range, 74.2% in a rice cooker, and 79.8% under pressure, with a concomitant increase in the degradation product protocatechuic acid [24]. Prolonged or severe heating may therefore deplete compounds that could otherwise contribute to digestive enzyme inhibition. Phenolics’ association with starch, including inclusion or non-inclusion complexation, may alter thermal and oxidative stability [75], but improved retention during processing does not necessarily guarantee release or inhibitory activity during gastrointestinal digestion. Studies claiming a phenolic-mediated digestibility effect should therefore quantify relevant compounds and degradation products before processing, after processing, and after simulated digestion, while distinguishing chemical retention from bioaccessibility.

## 6. Translating In Vitro to In Vivo: Low-Glycemic Index and Clinical Outcomes

### 6.1. Gaps Between In Vitro eGI and In Vivo Glycemic Index

Agreement between in vitro eGI and measured human GI is often imperfect because static digestion models do not capture gastric emptying, intestinal absorption, incretin responses, insulin action, and other physiological determinants of postprandial glycemia [83,87]. Peng et al. showed that for biscuits with clinically determined GI values, a specific 180 min reducing-sugar digestion metric predicted GI better than the INFOGEST protocol and required product-specific calibration [95]. At the individual level, glycemic responses to mixed meals also vary substantially; in a cohort of 800 participants, models incorporating clinical and gut microbiome variables improved prediction of person-specific postprandial responses compared with simple nutrient descriptors [96]. These findings do not invalidate standardized GI measurement as a group-level property of foods, but they show that in vitro eGI and individualized glycemic response prediction answer different questions. Estimated GI should therefore be used primarily for formulation screening or within-method ranking until validated against measured GI in humans.

### 6.2. International Standard for Glycemic Index Determination (ISO 26642:2010)

Formal classification of a food as low-, medium-, or high-GI requires measuring the postprandial glucose response in humans using a standardized protocol. ISO 26642 specifies a method that tests healthy participants after an overnight fast, provides a test portion containing a defined amount of available carbohydrate, and compares the resulting incremental glucose response with repeated reference-food tests [88]. Capillary blood sampling is commonly preferred because postprandial excursions are larger and, in GI methodology studies, generally less variable than venous responses [87,97]. The exact carbohydrate dose, number of participants, reference repetitions, sampling schedule, and data quality criteria should follow the laboratory’s current ISO method. The incremental area under the glucose response curve (iAUC) is calculated above the fasting baseline using the prescribed trapezoidal approach:

(5) $$\mathrm{iAUC}=\sum_{i=1}^{n}\frac{{\Delta C}_{i-1}+{\Delta C}_{i}}{2}\left(t_{i}-t_{i-1}\right),$$

where Δ*C_i_* denotes the glucose increment above the fasting concentration at time *t_i_*. Only incremental area above the fasting baseline contributes to iAUC; when the response crosses the baseline, the crossing point is determined by interpolation so that sub-baseline area is excluded. The participant-specific GI is then calculated as the ratio of test-food iAUC to the mean reference-food iAUC, multiplied by 100:

(6) $$\mathrm{GI}=\frac{{\mathrm{iAUC}}_{\mathrm{test food}}}{{\mathrm{iAUC}}_{\mathrm{reference}}}\times 100$$

GI is calculated for each participant and summarized across participants according to the standardized method. Foods are commonly classified as low (≤55), medium (56–69), or high (≥70) GI [89]. For products containing physically modified starch, two practical controls are especially important. First, measure the available-carbohydrate content used to determine the test portion in the final processed product using a method that appropriately accounts for resistant or non-available carbohydrate. Second, standardize production and storage conditions because retrogradation and moisture redistribution can continue after processing. In addition, GI should be interpreted as a standardized measure of the glycemic quality of available carbohydrate, not as a complete measure of serving-size glycemic load, nutrient density, or long-term clinical efficacy.

### 6.3. Clinical Evidence on Blood Glucose and Insulin Responses

A lower measured GI is generally associated with a smaller incremental glucose exposure and may also be accompanied by a reduced insulin response, although the magnitude and timing depend on food composition and participant characteristics. Matrix design can achieve substantial acute effects in staple foods. In healthy participants, replacing 30% or 60% of wheat flour in white bread rolls with cellular chickpea powder, in which starch remained partly encapsulated within intact cell wall structures, reduced glycemic iAUC by approximately 40% while maintaining acceptable sensory properties [98]. D-Allulose represents a different strategy based on co-ingestion rather than structural encapsulation: in a randomized crossover study of 30 adults without diabetes, 10 g of allulose consumed with a 50 g sucrose load reduced postprandial glucose and insulin excursions [99]. A meta-analysis likewise reported attenuation of postprandial glucose exposure at 5 and 10 g doses in healthy participants [100], whereas very small “catalytic” doses have not shown a consistent effect [101].

Human evidence differs substantially by intervention type. In individuals described as having borderline diabetes, allulose reduced the postprandial glucose response [93]. Resistant-starch supplementation has also been studied in insulin-resistant adults: 40 g/day RS for 12 weeks improved insulin sensitivity measured by euglycemic–hyperinsulinemic clamp without significant changes in body weight or ectopic fat [102], while an 8-week crossover trial using 40 g/day high-amylose maize RS2 increased forearm muscle glucose uptake and altered adipose-tissue function but did not improve all measured metabolic endpoints [103]. Additional trials have reported lower postprandial glucose with RS supplementation in overweight adults [104], and low-GI dietary patterns improve glycemic control in people with diabetes [6]. Closer to the processing strategies discussed in this review, hydrothermally processed parboiled rice produced lower postprandial glucose than white rice in healthy participants in a randomized crossover pilot study, although the reduction was not significant in participants with T2DM [105], and cold storage of cooked non-glutinous rice and millet reduced glucose iAUC by 24.4% and 29.5%, respectively, compared with freshly cooked rice [60]. A meta-analysis of randomized trials in people with T2DM or prediabetes found that RS1 and RS2 lowered postprandial glucose, whereas evidence for RS3–RS5 was insufficient [106], and a meta-analysis in adults with metabolic syndrome-related risks reported consistent effects on lipids and some anthropometric measures but heterogeneous effects on fasting insulin and insulin resistance [107]. These findings support the metabolic relevance of slowing carbohydrate availability, but they should not be interpreted as direct clinical evidence for foods produced by HMT, annealing, ultrasound, or related physical modifications. Human trials of the modified foods themselves remain a key evidence gap; the reported benefit of HMT rice is presently preclinical [41].

**Figure 4:**
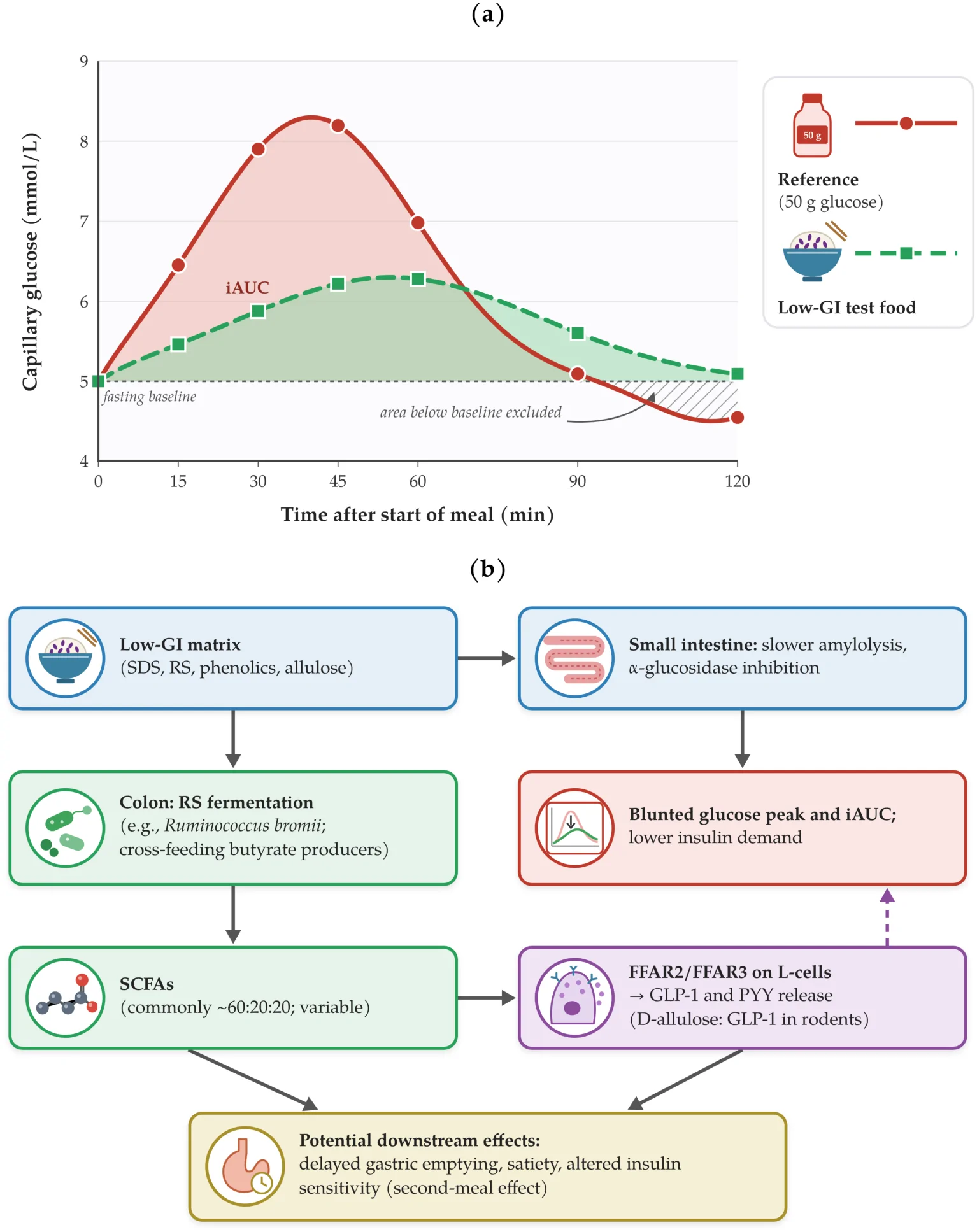
The physiological consequences of a low-GI food matrix. (**a**) Schematic postprandial capillary glucose responses to a 50 g glucose reference and a low-GI test food sampled at the time points specified in ISO 26642:2010 [88]; shaded areas represent the incremental area under the curve (iAUC), and hatched area below the fasting baseline is excluded from the calculation (curves are illustrative, not experimental data). (**b**) The proposed small-intestinal, colonic and endocrine pathways linking slowly digestible and resistant starch, phenolics and D-allulose to glycemic control [108,109,110,111,112,113,114,115,116]. SCFAs, short-chain fatty acids; FFAR, free fatty acid receptor; GLP-1, glucagon-like peptide-1; PYY, peptide YY.

The physiological consequences of resistant starch extend beyond small-intestinal glucose delivery. In the colon, specialist primary degraders such as *Ruminococcus bromii* can initiate degradation of particular resistant-starch structures [108], with subsequent cross-feeding to other microbial taxa; different RS types produce distinct microbiota responses [109]. Fermentation generates short-chain fatty acids (SCFAs), principally acetate, propionate, and butyrate. A ratio near 60:20:20 is often cited for human colonic SCFAs [110], but actual proportions vary with substrate, microbiota composition, colonic region, transit, and sampling matrix. SCFAs can activate free fatty acid receptors 2 and 3 (FFAR2 and FFAR3) on enteroendocrine and other host cells, and experimental studies support stimulation of glucagon-like peptide-1 (GLP-1) and peptide YY (PYY) secretion [111,112,113,114] (Figure 4). These pathways plausibly link fermentable carbohydrate structure to host metabolism, but the magnitude of their contribution to human glycemic outcomes remains context dependent. D-Allulose has also stimulated GLP-1 release and vagal signaling in mice [115], and in healthy Thai adults it lowered peak postprandial glucose and insulin after a sucrose load in a dose-dependent manner [116]; whether the mechanisms identified in animals, including inhibition of intestinal sucrase, operate at nutritionally relevant doses in humans requires further study.

### 6.4. Linking Starch Structure to Health Outcomes: Strength of Evidence

The evidence reviewed above can be arranged along a chain that runs from starch structure through in vitro digestion and acute human responses to long-term health outcomes (Table 6). The links are not equally strong. Many studies with direct structural measurements support the relationship between processing, structure, and in vitro digestibility, although mostly in isolated starches and model systems. The link between in vitro digestibility and acute human glycemic response is supported for some food categories, such as biscuits, but requires category-specific calibration [95] and has rarely been tested for physically modified foods. Short-term human evidence exists for cooled, parboiled, or cell wall-encapsulated staples and for RS supplements [60,98,102,105,106], whereas long-term outcomes such as glycated hemoglobin (HbA1c), insulin sensitivity, or diabetes incidence have been studied mainly for low-GI dietary patterns and RS supplementation rather than for specific clean-label products [5,6,102,103,107]. A product with lower in vitro digestibility should therefore be considered a candidate requiring human validation, not a demonstrated low-GI or health-promoting food.

## 7. Conclusions and Future Perspectives

### 7.1. Conclusions

Clean-label foods with reduced starch digestibility can be rationally designed by integrating raw material composition, physical processing, and food matrix engineering. HMT, annealing, ultrasound, extrusion, and controlled retrogradation can alter starch organization at multiple scales, while hydrocolloids, proteins, lipids, and phenolics can further modulate hydration, swelling, enzyme accessibility, and enzyme activity. Amylose–lipid V-type complexes provide a well-established RS5 mechanism, whereas starch–polyphenol interactions encompass both inclusion and non-inclusion structures whose classification and physiological relevance require case-specific characterization. The central principle is that glycemic response emerges from multiscale food structure rather than from crystallinity or amylose content alone. Operational measurements of RDS/SDS/RS, standardized gastrointestinal digestion models, kinetic parameters, and eGI calculations provide complementary screening information but are not interchangeable with measured human GI. Formal low-GI classification therefore requires in vivo validation, and longer-term clinical benefit requires evidence beyond the acute GI test. Across the framework, the evidence is strongest for structural effects of processing in isolated starches and flours, moderate for effects on in vitro digestibility in model systems, and weakest for effects of physically modified foods on human glycemic and long-term outcomes. Human trials of foods produced specifically by physical starch modification remain limited and should be prioritized.

### 7.2. Implementing the Framework and Research Priorities

The framework is intended as a structured decision support tool rather than a validated predictive model. Table 7 translates each tier into the principal design variable, the recommended analytical method for evaluating it, the evidence needed before drawing a conclusion, and the main limitation. In practice, conclusions should move up the tiers only when the evidence required at the lower tier has been obtained in the actual product. For example, a structural change documented by XRD, FTIR, or DSC in an isolated starch should be confirmed in the cooked and stored food before digestion data are interpreted, and an in vitro reduction in digestibility should be treated as a reason to conduct human GI testing rather than as a basis for a low-GI claim.

The approaches discussed in this review are at different stages of validation. HMT, AN, controlled retrogradation, and amylose–lipid complexation are experimentally established at the ingredient level but still require testing in finished foods and in humans. Ultrasound and microwave-assisted treatments require scale-up studies that report energy use, heating uniformity, and product quality. Starch–polyphenol systems require experiments that remove unbound polyphenols, characterize complex structure, and compare digestion with and without co-processing, followed by fermentation and human studies if RS5-like behavior is claimed [69]. Allulose–protein conjugates require the sequence of experiments outlined in Section 4.2, beginning with a demonstration that they affect starch digestion at all. For every approach, randomized human studies using ISO 26642 procedures, followed by longer-term trials with clinically relevant endpoints, are the decisive tests.

### 7.3. Future Translation: Precision Texture and Metabolic Nutrition

One illustrative translational application is the design of metabolically appropriate texture-modified foods for older adults. Aging is associated with increasing prevalence of impaired glucose tolerance, sarcopenia, reduced masticatory function, and oropharyngeal dysphagia, although these conditions do not necessarily occur together in every individual. Conventional texture modification through pureeing or thickening can reduce visual variety and, in some settings, contribute to lower food intake, making sensory quality and nutrient density important design considerations. The IDDSI framework provides standardized terminology and practical testing methods for texture-modified foods and thickened liquids [117].

Extrusion-based 3D food printing could integrate texture customization with control of carbohydrate digestibility. Printable food inks typically require shear-thinning flow, sufficient yield stress for shape retention, and viscoelastic properties that allow deposited layers to support subsequent layers [118,119]. For dysphagia applications, these rheological requirements must be balanced with softness, cohesiveness, particle size, and other properties relevant to the intended IDDSI level; successful printing alone does not establish swallowing safety. Hydrocolloid-containing vegetable formulations have been printed into structures that meet selected dysphagia texture criteria [120], and starch–hydrocolloid interactions may simultaneously influence digestibility. In printed wheat starch gels, amylose content and hydrogen-bonding interactions determined printing accuracy and the resulting IDDSI level [121], and in a randomized crossover trial, an oat paste that met IDDSI Level 3 criteria produced lower postprandial glucose when the oat flour had been roasted at 90–110 °C rather than 160 °C [94], showing that texture compliance and glycemic response can be addressed simultaneously. Future formulations should therefore optimize printing performance, IDDSI compliance, nutrient density, sensory acceptability, and starch digestion kinetics together, and should verify that cooking, printing, cooling, and storage do not reverse the structural features responsible for slower digestion. Any low-GI claim would still require human validation.

Beyond texture-modified foods, data-driven approaches may support both personalized nutrition and process optimization, although these applications require different datasets and model structures. Clinical variables, continuous glucose monitoring, and microbiome features can improve prediction of individual postprandial responses [96], whereas process–structure models would instead require detailed information on moisture, temperature–time history, mechanical energy, molecular organization, and digestion kinetics. Integrating these two levels is a future research opportunity, but only once robust process-to-digestibility relationships have been established. Agricultural by-products such as pigmented rice bran may also provide phenolic-rich ingredients for matrix engineering, but their use should be evaluated for batch variability, sensory impact, contaminant concentration, and actual complex-forming activity before sustainability or low-glycemic benefits are assumed.

## Figures and Tables

**Table 1:** Representative chemically modified food starches, their typical reagents, primary technological functions, and potential effects on starch digestibility.

Modified Starch (E-Number)	Typical Reagent(s)	Principal Technological Function	Potential Effect on Digestibility
Oxidized starch (E1404)	Sodium hypochlorite	Lower paste viscosity, higher clarity, film formation	Primarily used to modify processing functionality; effects on starch digestibility are generally limited and depend on the extent of oxidation
Acetylated starch (E1420)	Acetic anhydride; vinyl acetate	Reduced retrogradation; freeze–thaw stability	Acetylation may modestly reduce enzymatic hydrolysis at higher degrees of substitution, but digestibility reduction is not its primary technological purpose
Distarch phosphate (E1412)	Sodium trimetaphosphate; phosphorus oxychloride	Cross-linking; tolerance to heat, acid and shear	Cross-linking can restrict granule swelling and enzyme accessibility; highly cross-linked starches may exhibit substantial resistance to amylolysis
Acetylated distarch adipate (E1422)	Adipic and acetic anhydrides	Acid, heat and shear stability	Cross-linking may restrict granule swelling and reduce enzyme accessibility, although the magnitude of the effect depends on the degree of modification
Hydroxypropyl distarch phosphate (E1442)	Propylene oxide plus phosphate cross-linker	Stable viscosity; freeze–thaw stability	Hydroxypropyl substitution and cross-linking may reduce enzyme accessibility, but the effect on digestibility is formulation- and substitution-dependent
Starch sodium octenyl succinate (E1450)	Octenyl succinic anhydride	Emulsification; encapsulation	Primarily used as an amphiphilic emulsifying starch; partial resistance to amylolysis may occur but is not its principal functional role

Information compiled from [11,12].

**Table 4:** Food matrix components that modulate starch digestion: dominant mechanisms and representative evidence.

Matrix Component	Examples	Dominant Mechanism	Representative Evidence
Hydrocolloids	Xanthan, guar and locust bean gums, konjac glucomannan, pectin	Water competition; increased digesta viscosity; granule coating and network formation	Human: viscosity-dependent flattening of glucose response [62]; model systems: source-dependent reduction in hydrolysis [63] and RS up to 38.36% after HMT [38]
Proteins	Rice glutelin and prolamin; whey, soy and potato proteins	Physical barrier; limited swelling; partly lost on pepsinolysis	Flour: removal of endogenous protein and lipid increased rice flour digestibility [64]; model blends: potato starch–protein blends less digestible [65]
Maillard conjugates with D-allulose	Allulose–soy protein hydrolysate and allulose–pumpkin seed protein conjugates	Altered protein conformation and interfacial properties; hypothesized modification of starch accessibility	Demonstrated: conjugation modifies protein functionality [66,67,68]. Hypothesis: effects on starch digestion untested
Polyphenols and anthocyanins	C3G, P3G, tea polyphenols	Inclusion and non-inclusion complexes; mixed-type enzyme inhibition	Purified enzymes: C3G IC_50_ against pancreatic α-amylase 0.30 mM; synergy with acarbose [25]; isolated starch: tea polyphenol complex RS 56.25% [47]; RS5 assignment requires direct structural evidence [69]
Lipids	Free fatty acids, monoglycerides, emulsifiers	V-type amylose–lipid inclusion complexes (RS5)	Model systems: thermally stable complexes with reduced amylolysis [43,70]; oat flour: enzymatic extrusion increased RS [44]

Abbreviations: RS, resistant starch; HMT, heat–moisture treatment; C3G, cyanidin-3-O-glucoside; P3G, peonidin-3-O-glucoside; IC_50_, half-maximal inhibitory concentration; RS5, resistant starch type 5. The evidence source is indicated for each example; mechanistic interpretations are graded in the text (Section 1.4).

**Table 5:** Comparison of Englyst-type operational starch fractionation, INFOGEST 2.0 or adapted in vitro digestion models, and human glycemic index determination for evaluating lower-glycemic foods.

Parameter	Englyst-Type Operational Assay	INFOGEST 2.0/Adapted Digestion Model	Human GI Determination
Purpose	Operational classification of starch fractions	Simulation of gastrointestinal digestion and digestion kinetics	Determination of postprandial glycemic response
Primary outputs	RDS, SDS, RS	Digestion time course; extent of hydrolysis; kinetic parameters such as *k* and *C*_∞_	Glucose iAUC; GI
HI/eGI	Not intrinsic to Englyst classification	May be derived only when an additional validated hydrolysis index framework is applied	Not applicable
Physiological realism	Limited; operational assay	Oral–gastric–intestinal simulation but no absorption/hormonal feedback	Direct human response
Main use	Starch fraction characterization	Formulation screening and mechanistic comparison	Formal GI classification
Enzymes and key conditions	Pancreatic α-amylase and amyloglucosidase; fixed incubation times at 37 °C	Salivary α-amylase, pepsin, gastric lipase, pancreatin or individual enzymes, and bile; α-amylase activity should be measured separately [82]	Standardized available-carbohydrate dose; repeated reference-food tests; timed capillary sampling
Main sources of variability	Enzyme activity, sample preparation, particle size, and glucose assay	α-Amylase activity of pancreatin batches, substrate concentration, mixing, sampling times, and kinetic model	Between- and within-participant variation, reference food, sampling, and analytical precision
Comparability across studies	Comparable only under the same operational protocol	*k*, *C*_∞_, HI, and eGI comparable only when protocol, reference food, and regression equation are matched	Comparable when ISO 26642 or equivalent standardized procedures are followed
Relationship to other outputs	Cannot be derived from INFOGEST kinetic parameters	Cannot be converted into Englyst fractions; eGI requires calibration against human GI	Reference standard for GI classification

Compiled from [7,81,82,85,86,87,88,89]. RDS/SDS/RS and INFOGEST-derived kinetic parameters are complementary but methodologically distinct outcomes and should not be interpreted interchangeably.

**Table 6:** Evidence map linking processing and starch structure to in vitro digestion, short-term human responses, and long-term health outcomes, with the type of system from which the evidence derives.

Link in the Chain	Typical Evidence Source	Representative Examples	Strength of Evidence	Main Gap
Processing → starch structure	Isolated starches, flours	HMT, AN, ultrasound, microwave-assisted HMT, extrusion with lipids [32,35,37,44,45,46]	Strong for structural change (directly demonstrated); direction source- and condition-dependent	Few studies in finished foods after cooking and storage
Structure and matrix → in vitro digestibility	Isolated starches, model systems, some finished foods	Starch–hydrocolloid HMT systems; starch–polyphenol complexes; HMT barley noodles [38,39,47]	Moderate; mechanism often inferred from digestibility	Protocols and enzyme activities differ; complex formation and enzyme inhibition seldom separated
In vitro digestibility → acute human glycemic response	Finished foods tested in vitro and in humans	Biscuits with measured GI; parboiled and cooled rice; cellular chickpea bread [60,95,98,105]	Moderate for specific food categories; weak for physically modified foods	eGI rarely calibrated against measured human GI
Acute response → long-term metabolic outcomes	Human randomized controlled trials and meta-analyses	Low-GI dietary patterns; RS supplementation [5,6,102,103,106,107]	Moderate for dietary patterns and RS2; limited for RS3–RS5	No long-term trials of physically modified clean-label foods
Colonic fermentation and endocrine pathways	In vitro, animal, and human mechanistic studies	*R. bromii* degradation of RS; SCFA–FFAR2/3–GLP-1 signaling; allulose in rodents [108,109,110,111,112,113,114,115,116]	Mechanistically plausible; demonstrated mainly in models	Contribution to human glycemic control uncertain

Strength of evidence follows the classification described in Section 1.4. HMT, heat–moisture treatment; AN, annealing; RS, resistant starch; GI, glycemic index; eGI, estimated glycemic index; SCFA, short-chain fatty acid; FFAR, free fatty acid receptor; GLP-1, glucagon-like peptide-1.

**Table 7:** Implementation table for applying bottom–up framework: principal design variable, recommended analytical methods, evidence needed to support conclusion, and main limitation for each tier.

Tier	Principal Design Variable	Recommended Analytical Methods	Evidence Needed to Support a Conclusion	Main Limitation
1. Raw material and safety	Botanical source, amylose content, phenolic profile, contaminant levels	Starch and amylose analysis; HPLC of anthocyanins; ICP-MS with arsenic speciation; LC-MS/MS for pesticides and mycotoxins	Composition and contaminants within applicable limits for each batch, measured with validated methods	Batch and seasonal variability; enrichment of contaminants in bran
2. Physical processing	Moisture, temperature–time history, energy input, cooling and storage	XRD, ATR-FTIR, DSC, 13C CP/MAS NMR, microscopy; specific energy per kilogram	Reproducible structural change documented by orthogonal methods in the material that will be used	Structure–digestibility relationships are source-dependent; crystallinity is not a proxy for resistance
3. Food matrix interactions	Type and level of hydrocolloid, protein, lipid, phenolic compound, or allulose	Microscopy, rheology, swelling; free and bound phenolics; enzyme kinetic assays	Separation of reduced enzyme accessibility from direct enzyme inhibition; structural confirmation of complexes	Model system results may not persist after cooking, storage, or digestion
4A. Operational starch fractions	RDS, SDS, and RS profile	Englyst-type assay under stated conditions	Fractions measured in the finished food as consumed	Operational definitions; not equivalent to INFOGEST kinetics
4B. Digestion kinetics and eGI	*k*, *C*_∞_, HI, eGI	INFOGEST 2.0 or adapted protocol with measured α-amylase activity; first-order and LOS analysis	Same protocol, enzyme activity, reference food, and equation for all comparisons; calibration if eGI is reported	eGI is an estimate; no absorption or hormonal feedback
5. Human glycemic response	Available-carbohydrate dose, portion size, target population	ISO 26642 GI testing; insulin and incretin responses; CGM where appropriate	Measured GI in an adequately designed human study for a formal low-GI claim	Acute GI does not establish long-term benefit
6. Translation	Texture level, nutrient density, sensory acceptability, cost	IDDSI testing, sensory evaluation, shelf-life and techno-economic analysis	IDDSI compliance and retained digestibility profile after printing, cooling, and storage; long-term trials for health claims	Limited long-term evidence; individual responses vary

ATR-FTIR, attenuated total reflectance Fourier-transform infrared spectroscopy; CGM, continuous glucose monitoring; CP/MAS NMR, cross-polarization magic-angle spinning nuclear magnetic resonance; DSC, differential scanning calorimetry; HPLC, high-performance liquid chromatography; ICP-MS, inductively coupled plasma mass spectrometry; LC-MS/MS, liquid chromatography–tandem mass spectrometry; LOS, logarithm of slope; XRD, X-ray diffraction.

## Data Availability

No new data were created or analyzed in this study. Data sharing is not applicable to this article.

## Abbreviations

The following abbreviations are used in this manuscript:

AN	Annealing
ATR	Attenuated total reflectance
AUC	Area under the curve
C3G	Cyanidin-3-*O*-glucoside
CGM	Continuous glucose monitoring
CP/MAS NMR	Cross-polarization magic-angle spinning nuclear magnetic resonance
DSC	Differential scanning calorimetry
eGI	Estimated glycemic index
FFAR	Free fatty acid receptor
FTIR	Fourier transform infrared spectroscopy
GI	Glycemic index
GLP-1	Glucagon-like peptide-1
HbA1c	Glycated hemoglobin
HI	Hydrolysis index
HMT	Heat–moisture treatment
HPLC	High-performance liquid chromatography
iAUC	Incremental area under the curve
ICP-MS	Inductively coupled plasma mass spectrometry
IDDSI	International Dysphagia Diet Standardisation Initiative
IDF	International Diabetes Federation
LC-MS/MS	Liquid chromatography–tandem mass spectrometry
LOS	Logarithm of slope
NMR	Nuclear magnetic resonance
P3G	Peonidin-3-*O*-glucoside
PYY	Peptide YY
RDS	Rapidly digestible starch
RS	Resistant starch
SCFAs	Short-chain fatty acids
SDS	Slowly digestible starch
T2DM	Type 2 diabetes mellitus
US	Ultrasound
XRD	X-ray diffraction
